# How Prof. Burnstock’s enthusiasm supported P2 receptor research in Germany

**DOI:** 10.1007/s11302-021-09768-9

**Published:** 2021-02-04

**Authors:** Ralf Hausmann, Heike Franke, Günther Schmalzing

**Affiliations:** 1 Institute of Clinical Pharmacology, RWTH Aachen University, Wendlingweg 2, 52074 Aachen, Germany; 2 Rudolf Boehm Institute of Pharmacology and Toxicology, University of Leipzig, Leipzig, Germany

## The concept of purinergic signaling and discovery of purinergic receptors

The concept of “purinergic neurotransmission” was first proposed in 1972 by Geoffrey Burnstock [1]. Purinergic neurotransmission and the potent actions of extracellular ATP on many cell types implied the existence of membrane receptors for ATP, which were first called “purinergic receptors” by Geoffrey Burnstock in 1976 [2]. The fascinating history of the discovery of the seemingly unlikely existence of purinergic signaling was excitingly told by Geoffrey Burnstock himself [3]. Thanks to his pioneering work, extracellular purines are now accepted as important signaling molecules in numerous physiological and pathological processes. Accordingly, the role of the purine nucleotide ATP as a potent extracellular neurotransmitter and agonist of the P2 receptor family is firmly established [4].

## The German research group “Neuronal and glial P2 receptors - molecular basis and functional significance”

In the year 2005, Peter Illes from the University of Leipzig came up with the idea to set up a research group for P2 receptor research in Germany. Initiated by him—and achieved not least through his tireless efforts—eight individual project working groups joined the application process. The resulting proposal, entitled “Neuronal and glial P2 receptors - molecular basis and functional significance,” aimed at strengthening research in the field of purinergic signaling in Germany.

**Fig. 1:**
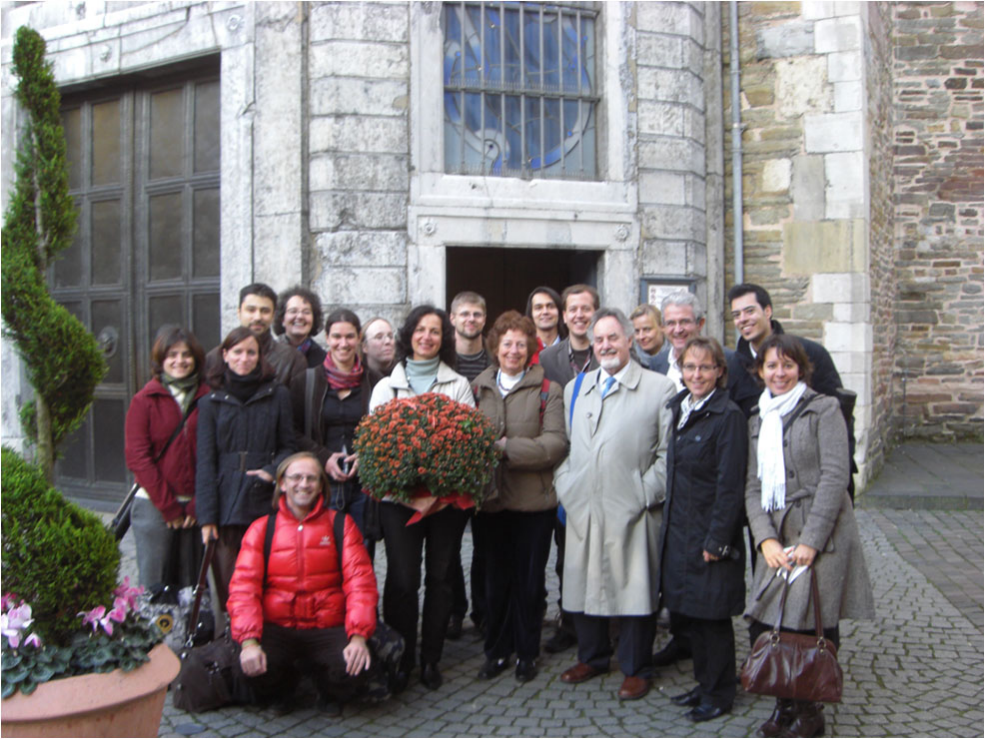
Group photo at the end of the Progress Report Meeting of the DFG Research Group *Neuronal and Glial P2 Receptors - Molecular Basis and Functional Significance* in 2008 in Aachen, Germany. The photo was taken in front of the main portal of the Aachen Cathedral. Prof. Geoffrey Burnstock and his wife Naomi are standing in the front row as 3rd and 4th persons from the right

As a leading expert in the field of purinergic signal transmission, Prof. Burnstock was invited by the German Research Foundation (DFG) to review our joint proposal to establish a national research group with the main research locations Leipzig, Frankfurt, and Aachen. The two-day review took place in 2006 in Leipzig in the presence of Geoffrey Burnstock and additional German experts invited by the DFG and members of the DFG itself. The original skepticism of the reviewers turned into agreement overnight. Judging by the few rumors that became known from the confidential nightly discussions of the reviewers, the enthusiastic support of Geoffrey Burnstock was decisive for the approval of our joint proposal FOR 748. The funding by the DFG was extended for another 3 years in 2010. Prof. Burnstock remained an interested guest and engaged discussion partner at numerous Progress Report meetings of the research group, e.g., in 2008 in Aachen (Fig. 1).

This reflects that, in addition to his own groundbreaking discoveries, Prof. Burnstock’s enthusiasm for research in purinergic signal transduction has also boosted P2 receptor research in Germany.

We thank Prof. Burnstock for his life’s work on purinergic signaling and his enthusiastic support of scientists in this field as well as the personal discussions and recommendations that have always enriched our research in the field of P2 receptors.

## References

[1] Burnstock G (1972). Purinergic nerves. Pharmacol Rev.

[2] Burnstock G (1976). Purinergic receptors. J Theor Biol.

[3] Burnstock G (2012). Purinergic signalling: Its unpopular beginning, its acceptance and its exciting future. Bioessays.

[4] Ralevic V, Burnstock G (1998). Receptors for purines and pyrimidines. Pharmacol Rev.

